# Seismic source analysis of the destructive earthquake November 21, 2022, M_w_ 5.6 Cianjur (Indonesia) from relocated aftershock

**DOI:** 10.1038/s41598-024-60408-9

**Published:** 2024-05-27

**Authors:** Zulfakriza Zulfakriza, Andri Dian Nugraha, Nova Heryandoko, Rexha Verdhora Ry, Faiz Muttaqy, Ade Andika, Muhammad Fikri Azhari, Ade Surya Putra, Kadek Hendrawan Palgunadi, Phil R. Cummins, Pepen Supendi, Aditya Lesmana, David P. Sahara, Nanang T. Puspito

**Affiliations:** 1 Global Geophysics Research Group, Faculty of Mining and Petroleum Engineering, Institut Teknologi Bandung, Bandung, 40132 Indonesia; 2 Research Center for Disaster Mitigation, Institut Teknologi Bandung, Bandung, 40132 Indonesia; 3 Indonesian Meteorological Climatological and Geophysical Agency, Jakarta, Indonesia; 4 Geophysical Engineering Study Program, Faculty of Mining and Petroleum Engineering, Institut Teknologi Bandung, Bandung, 40132 Indonesia; 5 Research Center for Geological Disaster, National Research and Innovation Agency (BRIN), Bandung, 40135 Indonesia; 6 Earth Science Study Program, Faculty of Earth Science and Technology, Institut Teknologi Bandung, Bandung, 40132 Indonesia; 7 Geophysical Engineering Department, Insitut Teknologi Sepuluh Nopember, Surabaya, Indonesia; 8 Swiss Seismological Service (SED), ETH Zürich, Zürich, Switzerland; 9 Research School of Earth Sciences, The Australian National University, Canberra, Australia; 10 Department of Earth Sciences – Bullard Labs, University of Cambridge, Cambridge, CB30EZ UK

**Keywords:** Seismology, Natural hazards, Geophysics

## Abstract

A destructive shallow earthquake with a magnitude of 5.6 struck Cianjur, West Java, Indonesia on November 21, 2022. This earthquake resulted in 602 casualties and the collapse of over 67,504 residences. The day after the mainshock, we deployed 19 temporary seismic stations to monitor aftershocks for a period of 30 days. We manually picked arrival times for 4499 P-waves and 3419 S-waves and determined locations for 514 events. Following the velocity model update, phase refinement through waveform cross correlation, and relocation using double-difference methods, we were able to determine 442 well-defined hypocenters of the aftershocks. We identified two clusters of aftershocks: one in the NNW-SSE direction, with a length of about 8 km, and another in the WSW-ENE direction, with a length of around 6 km. The seismogenic zone of these clusters ranges from a depth of 3 to 13 km. Our interpretation suggests that these clusters may indicate a conjugate fault. It is possible that the mainshock (Mw5.6) Cianjur earthquake on November 21, 2022 occurred on the WSW-ENE direction with sinistral movement.

## Introduction

A moderate magnitude, yet destructive, earthquake (Mw 5.6) occurred in Cianjur, West Java, Indonesia, at 13:21 local time (UTC + 7 h) on November 21, 2022 (later defined as the Cianjur earthquake). According to the Indonesian Agency for Meteorology, Climatology, and Geophysics/Badan Meteorologi, Klimatologi dan Geofisika (BMKG), this event occurred at a focal depth of approximately 11 km and involved horizontal movement along an active fault. The earthquake caused significant shaking in the Cianjur district, located approximately 102 km southwest of Jakarta the capital city of Indonesia. The most severe damage was observed in the Cugenang subdistrict, the nearest populated area to the epicenter, with an intensity rating of VIII on the Modified Mercalli Intensity (MMI) scale (https://earthquake.usgs.gov/earthquakes/eventpage/us7000ir9t/executive). The National Agency for Disaster Management of Indonesia/Badan Nasional Penanggulangan Bencana (BNPB) reported 602 casualties and 67,504 residences collapsed (https://gis.bnpb.go.id/Cianjur2022/).

**Figure 1:**
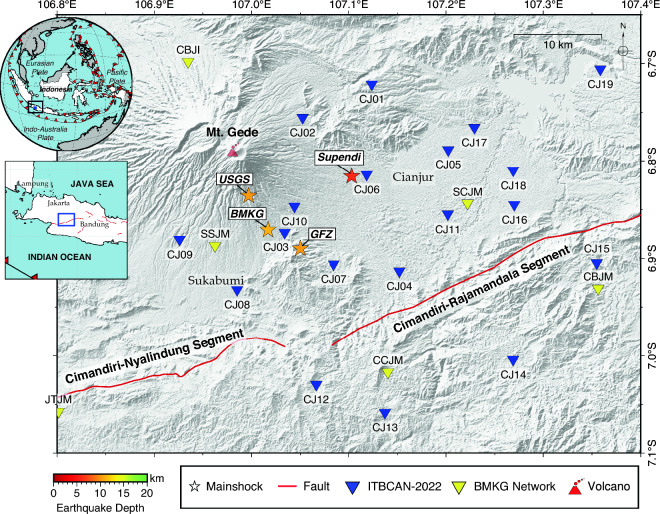
The mainshock epicenter of the Cianjur earthquake on November 21, 2022, as reported by three different institutions, is marked by an orange star. The relocated epicenter denoted by a red star was determined based on a previous study^7^. Distribution of ITBCAN temporary seismic stations (19 stations) marked by blue inverted triangle and BMKG permanent seismic stations marked by the yellow inverted triangle. The red lines identify the active fault lines^2^. The upper left inset map shows the location of West Java (black rectangle) with respect to Southeast Asia. The lowest left inset map shows the location of the study area (blue rectangle) with respect to West Java.

Tectonically, West Java and the surrounding area, including the Cianjur district, are influenced by Java subduction and active shallow crustal faults (Fig. 1). The convergence of the Australian and Sunda Plates at 67 mm/year^1^ is accommodated by subduction of the Australian Plate beneath Java, and this influences the activity of shallow crustal faults in West Java, such as the Cimandiri, Lembang, Garsela, and Baribis Faults^2,3^. According to the Indonesian earthquake catalogs between 1900 and 2016^2^, most of the earthquakes near the Cianjur district appear to cluster of the Cimandiri Fault Zone (CFZ) with left-lateral strike-slip faulting^4,5^. Based on historical records^6^, several destructive earthquakes occurred in Cianjur and the surrounding region, including events such as the October 10, 1834 with a maximum intensity of VIII, the February 15, 1844 with a maximum intensity of VIII, and the December 18, 1910 with a maximum intensity of VII. However, the causative faults remain unknown.

The Cianjur earthquake did not occur on the fault line that described by the National Center for Earthquake of Indonesia^2^ (Fig. 1). The Cianjur earthquake was probably generated by a hidden fault that mostly covered by recent volcanic deposits. Preliminary findings of the Cianjur earthquake using 13 permanent seismic stations in West Java operated by BMKG identified 196 relocated aftershocks between November 21, 2022 and January 8, 2023^7^. They discovered the presence of a conjugate fault pair trending NNW-SSE and another fault trending WSW-ENE direction^7^.

In this study, we further investigate by establishing a more comprehensive seismic network to understand seismic sources. We show the spatial distribution, magnitudes, and moment tensor of aftershocks of the Cianjur earthquake in order to identify the causative fault orientation. We deployed 19 temporary seismic stations for one month of recording, starting a day after the mainshock. In the following steps, we manually inspected waveform picks of P-wave and S-wave. The dense coverage of our local network allowed us to update the velocity model and conduct waveform cross correlation^8^ and hypoDD algorithms^9,10^ to relocate the aftershocks.

Our analysis focused on understanding the spatial distribution of relocated aftershocks to better comprehend their source mechanism since well-located aftershocks can be useful in determining fault plane orientations.

## Data and m﻿ethods

We deployed the local temporary seismic network soon after the mainshock Mw5.6 in Cianjur. The continuous seismic waveform recorded by three-component short-period node geophone belong to ITBCAN-2022 (Institut Teknologi Bandung for Cianjur Aftershock Network-2022). The ITBCAN-2022 used the SmartSolo IGU-16HR 3C with a natural frequency of 5.0 Hz and the signal was digitized at a sampling rate of 250 Hz. A total of 19 seismic stations were deployed, with 6 stations set up on November 22, 2022 (a day after the mainshock), and an additional 13 seismic stations added on November 26, 2023 (five days after the mainshock). Our seismic network across the epicentral area of 50 km × 55 km started recording the seismic activity from November 22 to December 24, 2022 (Fig. 1). Some seismic stations were deployed in the southern part to anticipate aftershocks occurring in both the Cimandiri Rajamandala segment and Cimandiri Nyalindung segment (see Fig. 1).

**Figure 2:**
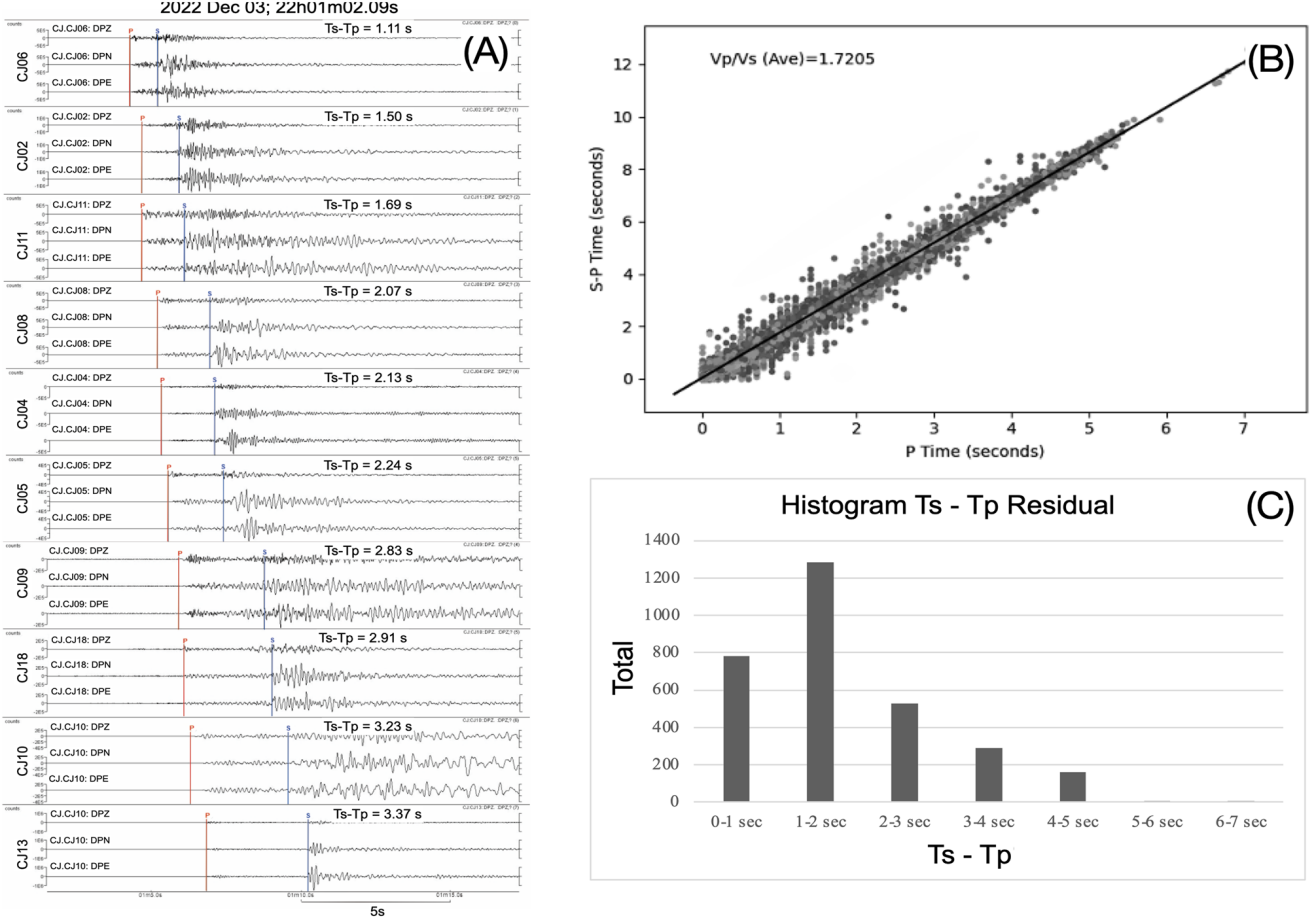
(**A**) 10 seismic stations recorded an event on 03 December 2022 using three-component seismogram, from top to bottom CJ06, CJ02, CJ11, CJ08, CJ04, CJ05, CJ09, CJ18, CJ10, and CJ13. Red and blue lines indicate the onset of P- and S- wave arrival times. (**B**) Wadati diagram of 514 events and its corresponding VP/Vs ratio of 1.7205. (**C**) Histogram of Ts-Tp residual for 514 events.

Visualization of the P- and S-wave arrival-time phases of each recorded event using the Seisgram2K^11^ as shown in Fig. 2A. We carefully selected only those events recorded by at least four stations for further studies. A total of 514 local earthquakes were detected by manually picking, with 4,499 P-wave and 3,419 S-wave picks (Fig. 2A). A Wadati plot was used to estimate the Vp/Vs ratio at about 1.72 (Fig. 2B). We subsequently identified the initial location of these local events using a Non-Linear Location (NonLinLoc) method^12,13^. The 1D seismic velocity model representing the study area was derived from an S-wave velocity (Vs) model of West Java^14^ with the P-wave velocity (Vp) directly calculated using a Vs-to-Vp scaling relationship^15^. The spatial location errors are provided in the confidence ellipsoids (68% probability) of the sampled posterior probability density function that is centered on the expectation hypocenters^16^.

We conducted spectral analyses on the three-component seismograms to determine the magnitude. The moment magnitude was determined by converting the earthquake's seismogram into displacement, and using the Fast Fourier Transform to calculate the spectra in the frequency domain. Then, we estimated the moment magnitude by fitting Brune point source spectral model to the observed spectra^18^,. We only analyzed the events with location errors of 5 km or less, and which were detected by a minimum of three stations. This resulted in 514 events for which moment magnitudes were calculated. The length of the P-wave time window we used is half of the S and P travel-time differences, and the S-wave time window is 1.5 times the length of the P-wave window. The maximum P-wave time window is 3 s. This length is sufficient as the events have epicentral distances of less than 50 km, and the time window must exclude later arriving phases.

**Figure 3:**
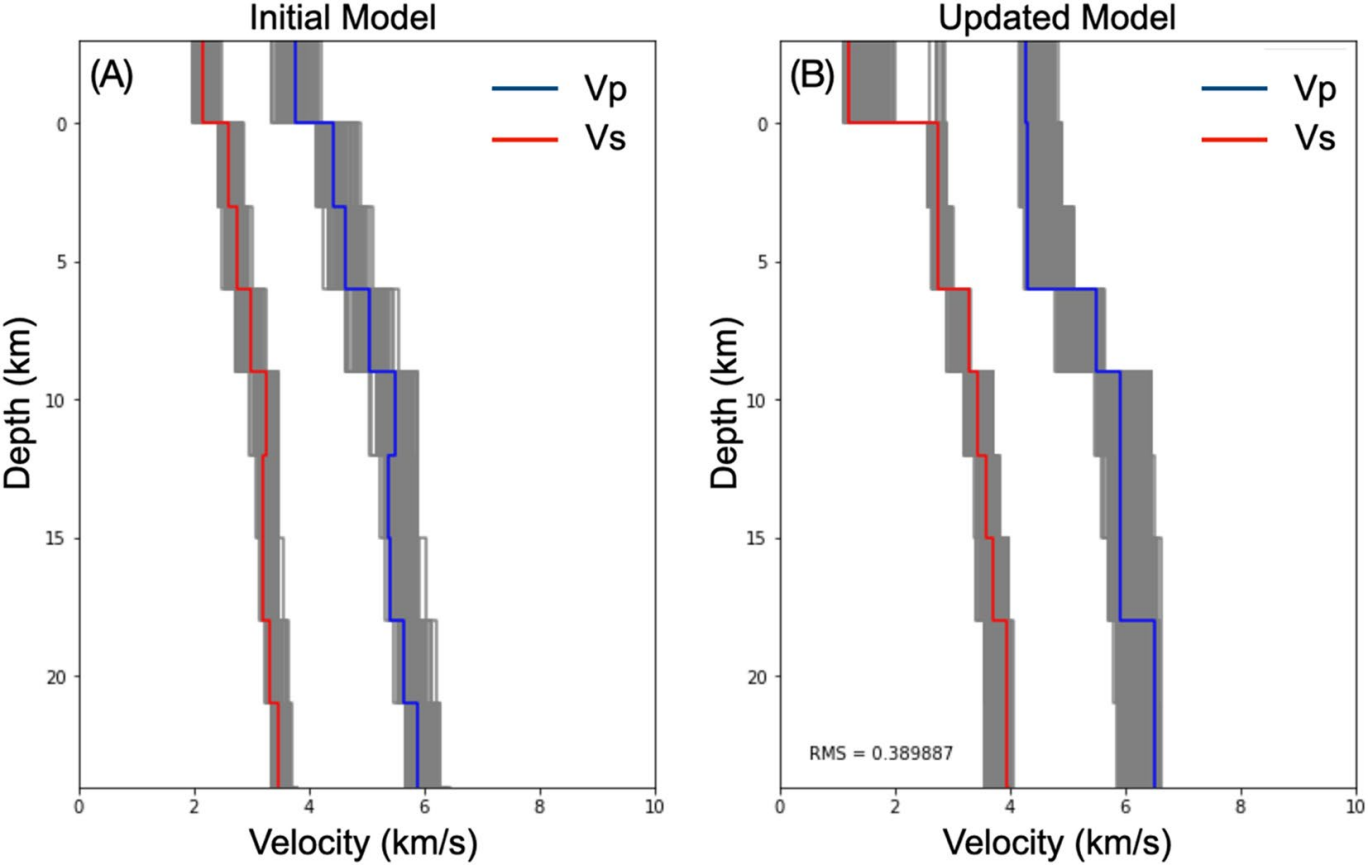
Profile of (**A**) 1-D initial models for P- and S-wave velocity, randomised from^12^ as a reference and (**B**) 1-D updated models. The final model (red for Vs and blue for Vp) is selected based on the minimum RMS residual of 0.389887 s.

To improve the accuracy of the earthquake catalog, we used VELEST^19^ and followed the procedure^20^ to invert both the 1-D velocity model and hypocenters that best fit the data. We utilized 514 earthquakes previously determined using NonLinLoc that encompassed data with an azimuthal gap < 180 and a minimum of six arrival picks. These criteria ensure high-quality events that are well-identified by our seismographic network. We selected CJ06 station as the reference station due to its central location for the aftershock distribution and a high number of event recordings. As the starting models, we generated 100 random models with a 3-km thickness layer, 10% perturbed from the 1-D velocity model used in the NonLinLoc analysis^15^ as the reference, to cover a wide range of plausible 1-D average structures around the study area. Including the reference model, these perturbations generated 101 initial models of Vp and Vs. The Vp/Vs ratio of 1.72 (based on the previous analysis of the Wadati diagram shown in Fig. 2B,C) was maintained for these generated perturbed velocity models. Then, we varied the damping values of 0.01 to 0.1 with a step of 0.01 (resulting in 10 damping values) and used them for each initial velocity model. This combination generated a set of 1010 (101 × 10) initial models. Using this set, for each initial model respectively, we simultaneously inverted for 1-D velocities (Vp and Vs) in VELEST using joint data of P and S phases. This resulted in 1010 updated Vp and Vs models. The final optimum 1-D velocity model was selected based on the minimum RMS residuals among all updated models, ranging from 0.39 to 0.52 s, and is indicated by the blue and red lines in Fig. 3B for Vp and Vs models, respectively. This final model was then used for hypocenter relocation using the double-difference method.

We improved the quality of P and S arrival times obtained from manual picks by performing a cross-correlation procedure with representative waveforms. A clear waveform with a high Signal-to-Noise Ratio (SNR) and high confidence in the arrival times of P and S waves was chosen as a reference event. We performed waveform cross-correlations (WCCs) at each single station between its reference event and each waveform of the other aftershocks, to determine the best time lags^8^. Then, the arrival times of the P and S waves obtained by manual picks were corrected using the best time lag resulting from the WCCs. The bandpass filter of 1 and 3 Hz for both the P wave and S wave with a time window of 0.05 s before P to 0.6 s after P and 0.1 s before S to 1.3 s after S was chosen. About 1588 of 4340 P-waves with a correlation coefficient ≥ 0.8, and 1539 of 3329 S-waves with a correlation coefficient ≥ 0.6 resulted from the WCCs. Arrival times were not adjusted for waveforms with correlation coefficients less than these thresholds. The 1-D velocity model obtained from VELEST and the arrival times of P and S wave obtained from WCC were combined with picked P and S-phases to improve the initial hypocenter locations resulting from NonLinLoc (e.g.^21^).

For our final hypocenter solutions, we used HypoDD^9,10^, which uses the double-difference (DD) algorithm that makes the assumption that the hypocenter separation between two earthquakes is small compared to the event-station distance and the scale length of velocity heterogeneity^10^. This method is appropriate for this study since the initial locations of the aftershock distribution lie in a tight cluster of 10 by 10 km^2^ wide. The least square method^22^ was used to solve the system of DD equations. The damping value of 60 was used for the first 3 iterations and a value of 50 for the subsequent 3 iterations (in total 6 iterations), with the weighting of the distance between paired events. We chose these parameter settings to avoid instability of hypocenter adjustments and to keep the value of the DD linear system condition number between the values 40–80 as suggested by^9^. The double-difference method has been successfully used to study earthquakes in Indonesia (e.g.^3,21^).

Earthquake moment tensor were determined using the full waveform inversion method implemented in ISOLA^23^. We used the local velocity model resulting from the VELEST analysis for the calculation of Green’s function waveforms. After removing the instrument response, the observed waveforms used for moment tensor inversion were high-pass filtered with corner frequencies of 0.15 Hz to 0.2 Hz. The quality of the moment tensor solution is indicated by high Variance Reduction (VR) and Double-Couple (DC) percentage with small Conditional Number (CN)^24^.

## Results and discussion

**Figure 4:**
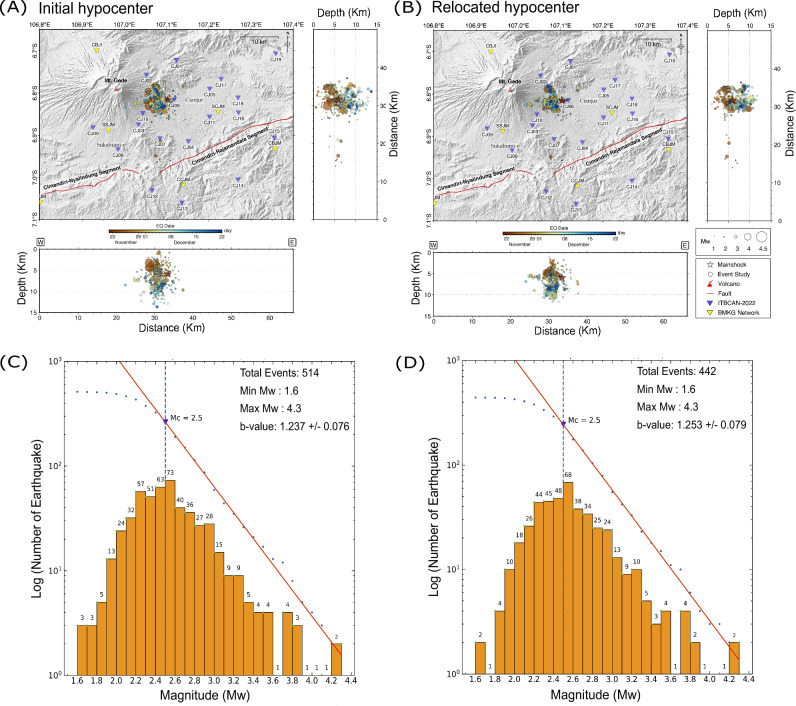
Map of shallow aftershocks (depth < 15 km) within a period of November 22, 2022, and December 24, 2022, the relocated mainshock^7^ marked by the red star. (**A**) 514 initial hypocenters of aftershocks. (**B**) 442 well-defined relocated hypocenters of aftershocks using HypoDD after waveform cross correlation and the shallow events mostly occurred at the beginning. Frequency Magnitude Distribution (FMD) with magnitude of completeness 2.5, before relocation (**C**) and after relocation (**D**).

**Figure 5:**
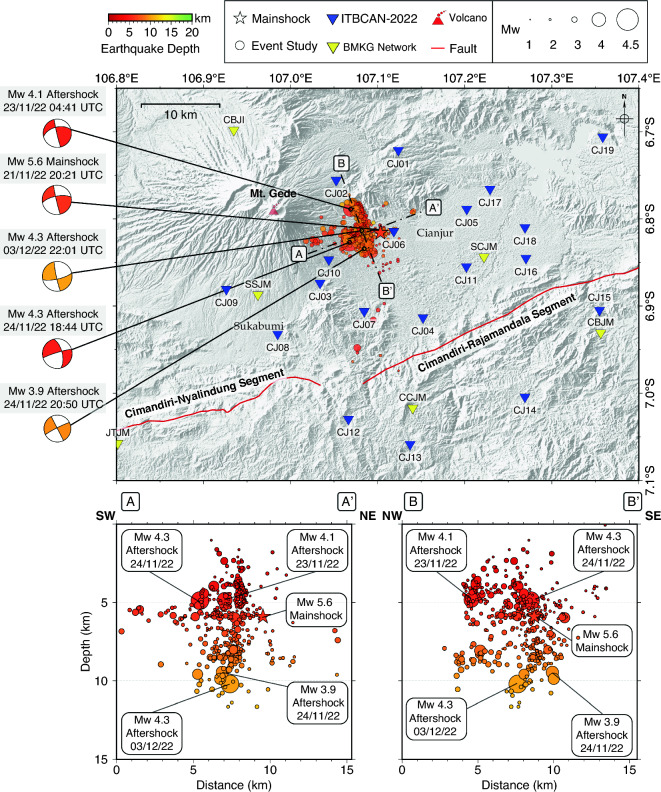
Map view of the relocated mainshock^7^, relocated aftershocks and moment tensor solution of the large aftershocks (M = 3.9 and above). The red star represents the relocated mainshock^7^ and the red to yellow circles illustrate the aftershocks. The A-A’ and B-B’ show vertical cross section along SW-NE and NW–SE direction, respectively.

We determined the 514 aftershock events of Cianjur earthquake by manual picking of 4,499 P- and 3,419 S-wave arrival times (Fig. 4A,C). By using the waveform cross-correlation (WCC) and hypoDD method, we successfully relocated 442 out of 514 events and the comparison of the relocated aftershock with their initial locations, as shown in Fig. 4B,D. The relative accuracy of the aftershock hypocenters was improved, and the directional distribution of the aftershock pattern was more clustered compared to the initial hypocenter locations. We also determined the moment tensors of significant larger aftershocks felt by the people, November 23, 2022, 04:41 UTC (Mw4.1), November 24, 2022, 18:44 UTC (Mw4.3), November 24, 2022, 20:50 UTC (Mw3.9) and December 3, 2022, 22:01 UTC (Mw4.3) as shown in Fig. 5.

Using temporary recordings advanced this study, increasing the resolution of seismic activity by capturing more events and revealing a clearer fault geometry (see Fig. 5), whereas a previous study using only permanent station data (Fig. 1, yellow triangles) showed a cloud of seismicity. This study confirms some conclusions from this previous study^7^, such as the presence of aftershocks on conjugate faults, with the mainshock located on a WSW-ENE oriented fault. We used the relocated mainshock^7^ and combined it with 442 relocated aftershocks (see Figs. 4B and 5). In general, the hypocenters are indicative of a fault geometry that dips perpendicularly which is also consistent with nodal planes from moment tensors of the largest aftershocks, as illustrated in Fig. 5. We demonstrated that the aftershock of the Cianjur earthquake were mainly concentrated at the western edge of the relocated mainshock^7^, approximately 20 km north of Cimandiri Rajamandala segment.

The relocated aftershocks extend across an area of approximately 10 square km, which is relatively dense following the moderate mainshock. Based on their locations, we propose that there are two main clusters of aftershocks distributed in the Cianjur area due to unidentified fault structures with WSW-ENE and NNW-SSE orientation. The shorter cluster has a WSW-ENE direction with a length of ~ 6 km. The mainshock most likely occurred near the shorter cluster with sinistral movement along a WSW-ENE direction fault line. And the second cluster has the NNW-SSE direction with length ~ 8 km and may aligns with the conjugate fault that static and dynamically triggered by the mainshock rupture. Two days after the mainshock on November 23, 2022, there was evidence of a felt aftershock event with a magnitude of Mw = 4.1, and its epicenter was located in the second cluster (see Fig. 5).

The depth of these relocated aftershocks is found to be shallower than 13 km. The distribution of seismicity delineates the seismogenic zone between depth of 3 to 13 km. The earlier relocated aftershocks occurred at shallower depths, while the later events were mostly deeper (see Fig. 4B). We conjecture this distribution as the fault rupture zone resulting from the Cianjur mainshock^7^. It should be noted that identifying any surface rupture is challenging due to either a layer of alluvial material or recently deposited volcanic material on top of the epicenter region. Therefore, the distribution of the aftershock sequence lies within the seismogenic zone located in the shallow crust.

During co-seismic phase of rupture, the potential strain energy is converted to fracture energy to sustained the rupture, heat energy dissipated during rupture, and radiated energy propagated with the seismic waves. The radiated energy also brings dynamic stress transfer (additional shear stress and normal stress clamping-unclamping) but it's quickly attenuated with 1/r^2^. Therefore, during co-seismic phase, if an earthquake is hosted by multifault segments, dynamic stress transfer has a significant contribution^25,26^. In addition, as rupture arrested at a given space and time during co-seismic phase, the slip on the arrested part of the fault transfers static stress to the neighboring fault(s) (e.g., static coulomb stress^7^). Therefore, during this event within the co-seismic phase, both mechanisms must occur at the same time.

According to the moment tensor for four aftershock with larger magnitudes (greater than Mw = 3.9); see Fig. 5. The event of Mw4.3 (November 24, 2022) approximately consistent with the Mw5.6 mainshock and have a P-axis of compression in the WSW-ENE direction. And the others significant aftershock event have slip on a fault oriented in the NNW-SSE. In contrast to this compression axis, the moment tensor of the West Java regional seismicity study^3^ shows a dominant W-E direction, especially in the CFZ which is thought to be a sinistral fault^4^. Based on this data, it is suggested that the mainshock (Mw5.6) Cianjur earthquake likely occurred on a conjugate fault structure with WSW-ENE direction and sinistral movement similar to the structural pattern of the CFZ. Additionally, dextral movement was observed in the second cluster with NNW-SSE direction.

During the temporary local seismic observation (November 22, 2022 to December 24, 2022), the seismic activity in the CFZ experienced relatively few events. Further investigation (e.g., earthquake source modelling and finite fault inversion) is still needed to describe the fault plane that triggered the Mw5.6 Cianjur earthquake (November 21, 2022).

## Conclusions

The seismic source of the Cianjur earthquake Mw5.6 successfully identified using the earthquake parameter of 442 relocated hypocenters. Our temporary seismic network can enhance the description of aftershock numbers and patterns compared to data from permanent seismic stations discussed in previous study^7^. The distribution of relocated aftershocks shows clusters in space and time and relatively dense following the moderate earthquake (Mw5.6), with the seismogenic zone between 3 and 13 km. Two clusters of aftershocks may indicate a conjugate fault in the Cianjur area, and the mainshock (Mw 5.6) occurred in the shorter fault with sinistral movement. The findings of this study could be utilized for future research in areas like seismic tomography and seismic hazard assessment in Cianjur, West Java, Indonesia.

## Data Availability

The catalog of relocated hypocenters from temporary seismic stations is available on Zenodo (DOI: 10.5281/zenodo.10894944).

## References

[1] Simons WJF (2007). A decade of GPS in Southeast Asia: Resolving Sundaland motion and boundaries. J. Geophys. Res..

[2] Irsyam M (2020). Development of the 2017 national seismic hazard maps of Indonesia. Earthq. Spectra.

[3] Supendi P (2018). Identification of active faults in West Java, Indonesia, based on earthquake hypocenter determination, relocation, and focal mechanism analysis. Geosci. Lett..

[4] Dardji N, Villemin T, Rampnoux JP (1994). Paleostresses and strike-slip movement: the Cimandiri Fault Zone, West Java, Indonesia. J. Southeast Asian Earth Sci..

[5] Abidin HZ (1994). Crustal deformation studies in Java (Indonesia) using GPS. J. Earthq. Tsunami.

[6] Martin SS, Cummins PR, Meltzner JM (2022). Gempa Nusantara: A database of 7380 macroseismic observations for 1200 historical earthquakes in Indonesia from 1546 to 1950. Bull. Seismol. Soc. Am..

[7] Supendi P (2023). A conjugate fault revealed by the destructive Mw 5.6 (November 21, 2022) Cianjur earthquake, West Java, Indonesia. J. Asian Earth Sci..

[8] Mori J (2008). Determination of dip direction for the 2007 Chuetsu-oki earthquake from relocation of aftershocks using arrival times determined by cross-correlation. Earth Planet Sp..

[9] Waldhauser, F. hypoDD: A program to compute double-difference hypocenter locations, USGS Open-File Report, 01-113 (2001).

[10] Waldhauser F, Ellsworth WL (2000). A double-difference earthquake location algorithm: Method and application to the Northern Hayward fault. Bull. Seismol. Soc. Am..

[11] Lomax A, Michelini A (2009). Mwpd: A duration-amplitude procedure for rapid determination of earthquake magnitude and tsunamigenic potential from P waveforms. Geophys. J. Int..

[12] Lomax A, Thurber CH, Rabinowitz N (2000). Probabilistic earthquake location in 3D and layered models. Advances in Seismic Event Location. Modern Approaches in Geophysics.

[13] Lomax A, Michelini A, Curtis A, Meyers R (2014). Earthquake location, direct, global-search methods. Encyclopedia of Complexity and Systems Science.

[14] Rosalia S (2022). Upper crustal shear-wave velocity structure Beneath Western Java, Indonesia from seismic ambient noise tomography. Geosci. Lett..

[15] Brocher TM (2005). Empirical relations between elastic wavespeeds and density in the Earth’s crust. Bull. Seismol. Soc. Am..

[16] Press WH (1992). Numerical Recipes in C: The Art of Scientific Computing or Numerical Recipes in FORTRAN: The Art of Scientific Computing.

[17] Ottemoller L (2003). Moment magnitude determination for local and regional earthquakes based on source spectra. Bull. Seismol. Soc. Am..

[18] Stork AL, Verdon JP, Kendall J-M (2014). The robustness of seismic moment and magnitudes estimated using spectral analysis. Geophys. Prospect..

[19] Kissling E (1994). Initial reference models in local earthquake tomography. J. Geophys. Res. Solid Earth.

[20] Mousavi S (2015). Seismic tomography reveals a mid-crustal intrusive body, fluid pathways and their relation to the earthquake swarms in West Bohemia/Vogtland. Geophys. J. Int..

[21] Muttaqy F (2023). Double-difference earthquake relocation using waveform cross-correlation in Central and East Java, Indonesia. Geosci. Lett..

[22] Paige CC, Saunders MA (1982). LSQR: Sparse linear equations and least squares problems. ACM Trans. Math. Softw..

[23] Sokos EN, Zahradnik J (2008). ISOLA a Fortran code and a Matlab GUI to perform multiple-point source inversion of seismic data. Comput. Geosci..

[24] Sokos EN, Zahradnik J (2013). Evaluating centroid-moment-tensor uncertainty in the new version of ISOLA software. Seismol. Res. Lett..

[25] Palgunadi KH (2020). Dynamic fault interaction during a fluid-injection-induced earthquake: The 2017 M w 5.5 Pohang event. Bull. Seismol. Soc. Am..

[26] Palgunadi KH (2024). Rupture dynamics of cascading earthquakes in a multiscale fracture network. J. Geophys. Res. Solid Earth.

[27] Tian, D., *et al*. PyGMT: A Python interface for the Generic Mapping Tools (v0.11.0). Zenodo. 10.5281/zenodo.10578540 (2024).

